# Long-Term Use of Nitrous Oxide Resulting in Vitamin B12 Deficiency Causing Cervical Myelopathy

**DOI:** 10.7759/cureus.64046

**Published:** 2024-07-07

**Authors:** Ravi Kumar, Kanchan Devi, Farah Zahra, Antonio Galindo, Deepak Kumar

**Affiliations:** 1 Internal Medicine, Northwestern Medicine McHenry Hospital, McHenry, USA; 2 Internal Medicine, Rosalind Franklin University of Medicine and Science, North Chicago, USA; 3 Internal Medicine, The Brooklyn Hospital Center, New York, USA; 4 Internal Medicine, Jinnah Sindh Medical University, Karachi, PAK

**Keywords:** vit b12 deficiency, laughing gas, nitrous oxide toxicity, nitrous oxide misuse neurological manifestation myelitis vitamin b12 deficiency, nitrous oxide myelopathy

## Abstract

Nitrous oxide, commonly known as laughing gas, gained popularity in the medical field as an anesthetic. It also causes euphoria and acts as an anxiolytic in the human body. However, there is limited information available on its toxicity, particularly its neurotoxicity, which has been emerging in younger populations using it for recreational purposes. Here, we present a case of a young patient with subacute combined degeneration secondary to vitamin B12 deficiency from chronic use of nitrous oxide.

## Introduction

Nitrous oxide is a non-flammable, colorless, and odorless gas, also known as laughing gas. Initially discovered in the 1700s, it gained popularity in medicine due to its anxiolytic and anesthetic properties [1,2]. Despite its early use, advancements in medicine and the development of newer anesthetic agents have led to a decline in its medical use over the decades.

However, nitrous oxide abuse has become prevalent among the younger population, primarily because it induces a euphoric state and is readily available through whippet canisters. Additionally, it does not typically appear on standard drug screenings [3]. Chronic use of nitrous oxide has been linked to vitamin B12 deficiency, which can result in significant neurological damage [1].

## Case presentation

A 28-year-old healthy male with a known history of polysubstance use, including cocaine and daily nitrous oxide (up to 1 liter daily) for almost 15 years, presented to the emergency department with chief complaints of bilateral numbness in his hands and feet and an inability to walk properly with an ataxic gait for the past five days. A week prior to the onset of symptoms, he was involved in a motor vehicle accident (MVA), during which he did not experience any symptoms. Initially, the patient attributed his symptoms to the MVA, but due to severe progression, he decided to come to the emergency department (ED).

He denied muscle weakness, fever, chills, recent travel history, any known tick bites, outdoor activities, urinary or fecal incontinence, bladder obstruction, blurry vision, or changes in weight or appetite. He reported using cocaine one month ago, and his last use of nitrous oxide was two days before presenting to the emergency department. The rest of his family, medication, and social history, including alcohol and smoking were unremarkable.

The general physical examination was unremarkable. On neurological examination, he was alert and oriented to time, place, and situation, with intact memory and fund of knowledge. Naming and repetition were intact, he was fluent, and he followed commands well. Cranial nerves II-XII were intact. Bilateral lower extremities demonstrated loss of muscle bulk, but tone and strength were intact. There was a loss of proprioception and vibration sensation in both hands and feet, but the pinprick sensation was intact. Deep tendon reflexes were absent in the patellar and ankle regions. He was mildly dysmetric bilaterally on the finger-to-nose and heel-to-shin tests. He had no tremors and was found to have an ataxic gait.

**Figure 1:**
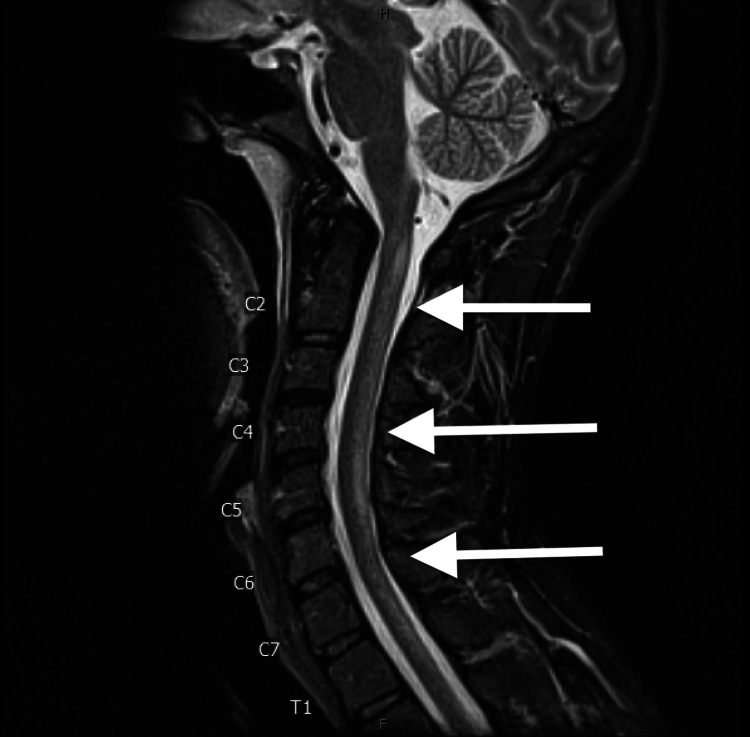
MRI of the cervical spine Moderate increased T2 signal intensity is seen from C4 to C5-C6.  Superiorly, this involves the central cord.  More caudally, it expands to the posterior cord on each side of the midline, slightly greater on the left than right.  Another cord signal abnormality is seen dorsally at C1.  Demyelination or inflammation would be favored.

Laboratory work, including complete blood count (CBC), comprehensive metabolic panel (CMP), lipid profile, and serum creatine kinase (CK) levels, were within normal limits. The urine drug screen was negative. Vitamin B12 was found to be low with a level of 164 pg/mL. Methylmalonic acid (2786 nmol/L) and homocysteine (108 µmol/L) levels were found to be very high. Tests for rapid plasma reagin (RPR), HIV antigen and antibody, and serum copper levels were negative. CT head and cervical spine without contrast were normal. MRI of the brain was normal, but MRI of the cervical spine showed cord signal abnormality at C1 and from C4 to C5-6, most likely due to inflammation or demyelination (Figure 1). MRI of the thoracic and lumbar spine and CT head and neck angiogram were negative for any abnormalities. The findings were suggestive of subacute combined degeneration secondary to vitamin B12 deficiency from chronic use of nitrous oxide.

The patient was started on vitamin B12 supplements with daily intramuscular (IM) injections and began physical therapy. He slowly showed improvement and was discharged to inpatient rehabilitation. He was also counseled on lifelong abstinence from nitrous oxide inhalation. The patient followed outpatient at the neurology clinic with complete resolution of symptoms after six months.

## Discussion

Nitrous oxide is now easily available, and there has been an increased trend among the younger population for recreational use. It is easily sold in whippet canisters online and is more commonly used through pre-filled balloons [4]. According to the American College of Emergency Physicians Toxicology Section, inhalant abuse in the United States is most common among adolescents aged 12 to 17, and its prevalence continues to increase. In 2019, 3.0% of adolescents and 1.7% of adults aged 18 to 25 reported using inhalants within the past year [5].

The prevalence of nitrous oxide use is increasing mainly due to its euphoric effects. The younger generation is often unaware of the potential toxic effects of chronic use. The most commonly known mechanism through which nitrous oxide causes neurotoxicity is by converting active vitamin B12 into its inactive form. Nitrous oxide oxidizes the cobalt ion of vitamin B12, which irreversibly inactivates cobalamin. Inactivated cobalamin cannot function as a coenzyme [3]. Methylcobalamin, a form of vitamin B12, is a cofactor that converts homocysteine to methionine. S-adenosylmethionine, a metabolite of methionine, is irreplaceable for the formation and maintenance of myelin sheaths. Vitamin B12 deficiency leads to impaired myelin synthesis and methylation of myelin proteins, resulting in neural demyelination [6-8].

The most common presentation is subacute combined degeneration, which involves the dorsal and lateral white columns, resulting in the loss of proprioception and vibratory sensation [9]. In our case, the patient experienced both sensory symptoms and ataxia. Usually, diagnosis is confirmed with an MRI of the spine and low levels of B12.

It is important to obtain a social history from young patients presenting with such neurological symptoms. Early recognition leads to better diagnosis and treatment, which usually involves replacing vitamin B12 levels and abstinence from nitrous oxide use. Patients often require extensive rehabilitation to achieve neurological recovery, which in our patient took four weeks of rehabilitation.

## Conclusions

Young patients presenting with acute neurological symptoms, including weakness and sensory disturbances, should always raise suspicion of nitrous oxide use. This can be confirmed with a detailed social history. The presentation can be further supported by low vitamin B12 levels. The purpose of writing this case report is to increase awareness of the chronic use of nitrous oxide leading to subacute combined degeneration. Prevention can be achieved by educating the younger population about its harmful impacts and encouraging complete abstinence.

## References

[1] Campdesuner V, Teklie Y, Alkayali T, Pierce D, George J (2020). Nitrous oxide-induced vitamin B12 deficiency resulting in myelopathy. Cureus.

[2] Nadal Bosch J, Malcolm J, Moya M, Menowsky M, Cruz RA (2023). A case report of subacute combined degeneration due to nitrous oxide-induced vitamin B12 deficiency. Cureus.

[3] Agarwal P, Khor SY, Do S, Charles L, Tikaria R (2021). Recreational nitrous oxide-induced subacute combined degeneration of the spinal cord. Cureus.

[4] Randhawa G, Bodenham A (2016). The increasing recreational use of nitrous oxide: history revisited. Br J Anaesth.

[5] (2024). Nitrous Oxide Misuse and Abuse. https://www.acep.org/toxicology/newsroom/jun2021/nitrous-oxide-misuse-and-abuse.

[6] Wu H, Huang H, Xu L, Ji N, Zhou X, Xie K (2023). Case report: subacute combined degeneration of the spinal cord due to nitrous oxide abuse. Front Neurol.

[7] Zheng D, Ba F, Bi G, Guo Y, Gao Y, Li W (2020). The sharp rise of neurological disorders associated with recreational nitrous oxide use in China: a single-center experience and a brief review of Chinese literature. J Neurol.

[8] Froese DS, Fowler B, Baumgartner MR (2019). Vitamin B(12), folate, and the methionine remethylation cycle-biochemistry, pathways, and regulation. J Inherit Metab Dis.

[9] Gürsoy AE, Kolukısa M, Babacan-Yıldız G, Celebi A (2013). Subacute combined degeneration of the spinal cord due to different etiologies and improvement of MRI findings. Case Rep Neurol Med.

